# Emergency department’s patient safety culture perceived by healthcare workers: A scoping review protocol

**DOI:** 10.1371/journal.pone.0325049

**Published:** 2025-05-27

**Authors:** Min Ji Kim

**Affiliations:** Department of Medical Humanities and Ethics, Hanyang University College of Medicine, Seoul, Republic of Korea; King Saud University / Zagazig University, EGYPT

## Abstract

A strong patient safety culture is critical for ensuring effective healthcare systems, particularly in high-risk environments such as emergency departments. Assessing patient safety culture requires the identification of strengths and weaknesses within clinical departments to enable targeted improvement. Patient safety in emergency departments is especially vulnerable due to overcrowding, necessity for rapid decision-making, and high pressure. However, the existing literature has not been systematically mapped to understand how healthcare workers perceive the patient safety culture in these settings. This scoping review aims to synthesize and map available evidence on patient safety culture as perceived by healthcare workers in emergency departments. This review will be conducted following the Joanna Briggs Institute methodology designed explicitly for scoping reviews, and the results will be reported following the Preferred Reporting Items for Systematic Reviews and Meta-Analysis extension for Scoping Reviews (PRISMA-ScR). The inclusion criteria will be based on the Population (healthcare workers), Concept (patient safety culture), and Context (emergency department settings) framework. A comprehensive search will be conducted in PubMed, CINAHL (EBSCOhost), Web of Science, Embase, Cochrane Library, KISS, and grey literature sources, such as ProQuest Dissertation & Theses Global and Google Scholar. Study selection and data extraction will be performed independently by two researchers, with a third researcher resolving discrepancies. Descriptive analysis will summarize the study characteristics, while content and thematic analyses will identify key themes related to patient safety culture. The findings will be presented at academic conferences and published in a peer-reviewed journal.

## Introduction

Ensuring patient safety is a fundamental priority in healthcare; however, avoidable medical harm remains a global challenge. The World Health Organization defines patient safety as the minimization of preventable healthcare-related harm through structured interventions [1]. The organization also emphasizes the importance of proactive strategies to mitigate the potential risks associated with patient safety incidents [1]. Key measures to realize patient safety include strengthening the patient safety culture, enhancing organizational policies, and ensuring comprehensive staff training and preparedness [2]. Rather than solely focusing on establishing new systems, there is greater emphasis on fostering a safety-oriented culture within clinical environments as this culture forms the essential foundation that enables these systems to operate effectively [3].

A strong patient safety culture fosters teamwork, open communication, and willingness to report and learn from safety incidents. By shaping the attitudes and behaviors of healthcare workers (HCWs), a well-established safety culture contributes to improved patient outcomes [4]. It also requires collaborative partnership and support from institutional management, which helps establish a strong foundation for patient safety [5]. Additionally, patient safety culture plays a crucial role in preventing patient safety incidents, as studies have shown that its implementation reduces such incidents in hospital settings [6].

In hospital settings, patient safety in emergency departments (EDs) is particularly vulnerable owing to several factors that compound the issue. First, overcrowding, referring to the state in which the ED operates beyond its optimal capacity [7], amplifies vulnerabilities by delaying time-sensitive interventions, treating patients in non-designated spaces, such as hallways, and increasing inpatient mortality [8]. Second, the triage process, a vital initial assessment, is prone to errors owing to multiple factors in high-pressure ED environments, including overwhelming patient volumes, diverse cases requiring rapid prioritization, and time constraints. Triage nurses face cognitive overload and fatigue, with accuracy rates varying significantly between novice and experienced nurses [9]. These challenges, combined with communication breakdowns and resource limitations, can lead to delayed treatment, misdiagnosis, and improper resource allocation [10]. Third, diagnostic errors are common, often resulting from insufficient assessment or miscommunication, and can lead to severe patient harm or even death [11]. Moreover, patients visiting the ED usually lack comprehensive medical records, making it difficult to track their medical histories, and they are sometimes unable to accurately describe their symptoms because of acute or severe illnesses [12]. These factors underscore the need for a robust patient safety culture to reduce and safeguard patient safety in the ED. This need is supported by a national cross-sectional study in the United States that found a negative correlation between the level of patient safety culture and the incidence of near-miss events [13].

Assessing the status of patient safety culture is a foundational step in identifying the strengths and weaknesses of developing tailored interventions that can establish a strong patient safety culture in the ED [14]. One of the most widely used tools for measuring patient safety culture is the Hospital Survey on Patient Safety Culture, developed by the United States Agency for Healthcare Research and Quality [15]. This survey assesses patient safety culture by identifying factors that shape attitudes and behaviors considered acceptable, expected, supported, and rewarded regarding patient safety. It covers 10 domains: (1) communication about errors, (2) communication openness, (3) handoffs and information exchange, (4) hospital management support for patient safety, (5) organizational learning (continuous improvement), (6) reporting patient safety incidents, (7) response to errors, (8) staffing and work-pace, (9) supervisor/manager/clinical leader support for patient safety, and (10) teamwork [15]. Another tool, the Safety Attitudes Questionnaire, has been adapted from an aviation industry questionnaire to measure management culture [16]. This questionnaire gathers perspectives based on six analytically derived factors: (1) teamwork climate, (2) safety climate, (3) job satisfaction, (4) perceptions of management, (5) working conditions, and (6) stress recognition [16].

*Patient safety culture* has been defined as the collective values, beliefs, and norms shared by frontline healthcare providers and nonclinical staff within an organization, which shape behaviors and reflect how an organization's culture promotes and supports patient safety [17]. Meanwhile, the *patient safety climate*, often viewed as a snapshot of patient safety culture, focuses on observable behaviors and attitudes related to safety [18]. While these terms are used interchangeably in the literature, in this review, we have unified them as patient safety cultures. This scoping review will consider studies that include HCWs. Here, *healthcare professionals* refer to licensed and trained individuals, typically doctors, nurses, or others, who provide direct patient care [19]. By contrast, *HCWs* encompass all healthcare staff, including clinical and non-clinical roles, such as quality improvement specialists, social workers, and nursing administrative managers [19]. Therefore, this review will consider HCWs as participants and include all the staff involved in healthcare settings.

Efforts have been made to examine how HCWs perceive patient safety culture in hospital settings since the development of instruments, such as the Hospital Survey on Patient Safety Culture. A preliminary investigation of PubMed, Web of Science, Google Scholar, and the Cochrane Database of Systematic Reviews revealed these efforts. For example, regional reviews focusing on Iran [20] and Latin America [21] have been conducted on the aspects of patient safety culture. Several reviews have explored patient safety culture in diverse healthcare settings, including pharmacies [22], nursing homes [23], operating rooms [24], and maternity units [25].

However, no existing in-progress scoping or systematic review has explicitly addressed the patient safety culture of HCWs in EDs. Regarding patient safety culture, both internal and external environmental factors, such as operational efficacy [26] and working conditions [27], significantly influence organizational culture by shaping shared values and practices. It is meaningful to map the concept of patient safety culture of EDs whose members have their values and practices, because unique factors of EDs that are different from those of other departments influence them.

Given these challenges, a comprehensive mapping of the literature is needed to understand how ED healthcare workers perceive the patient safety culture. This scoping review will identify research gaps and inform strategies for strengthening the patient safety culture in emergency care settings [28]. The protocol must first be completed to ensure that the review is conducted systematically and efficiently [29].

### Aims

The aims of the proposed scoping review are as follows:

(1) To assess the scope, nature, and extent of the literature on patient safety culture as described in existing literature perceived by HCWs in EDs
(2) To map the key concept related to patient safety culture perceived by HCWs in EDs
(3) To identify gaps in the literature and highlight areas for future research on patient safety culture in EDs.

## Materials and methods

The scoping review will follow the Joanna Briggs Institute (JBI)'s methodology designed explicitly for scoping reviews [30], which consists of five stages:

(1) Stage 1: Identifying the review questions—Defining the scope and objectives of the review.
(2) Stage 2: Identifying relevant studies—Developing a comprehensive search strategy based on the Population-Concept-Context framework.
(3) Stage 3: Study selection—Screening titles, abstracts, and full texts against predefined inclusion and exclusion criteria.
(4) Stage 4: Charting the data—Extracting relevant information from selected studies using a standardized data extraction tool.
(5) Stage 5: Collating, summarizing, and reporting results—Analyzing the extracted data through descriptive, content, and thematic analyses.

The full scoping review will be reported in accordance with the PRISMA extension for Scoping Reviews (PRISMA-ScR) (S1 File) [31].

### Stage 1: Identifying the review questions

Scoping reviews involve interactive refinement of review questions to ensure alignment with emerging evidence and research objectives [30]. The primary review question guiding this study is, *“What is known about patient safety culture as perceived by HCWs in EDs?”*

### Stage 2: Identifying relevant studies

The eligibility criteria for this review will follow the Population, Concept, and Context mnemonic recommended by the JBI for a scoping review [30].

#### Population.

This review will include studies examining HCWs in ED settings. HCWs will be broadly defined to include both clinical (e.g., physicians and nurses) and non-clinical staff (e.g., quality improvement officers and administrative managers) to provide a comprehensive perspective on patient safety culture in EDs.

#### Concept.

Studies focusing on patient safety culture, including its measurement, influencing factors, and organizational impact, will be included. Research addressing broader organizational culture without an explicit reference to patient safety culture will be excluded to maintain a focused scope.

#### Context.

Only studies conducted in ED settings within hospitals will be included. Research conducted in non-hospital settings (e.g., urgent care centers and ambulance services) will be excluded to ensure consistency in the contextual factors.

#### Exclusion criteria.

We will exclude studies that were not presented in English or Korean. While language restrictions can limit the comprehensiveness of reviews, research has shown that excluding non-English publications from systematic reviews of clinical interventions has minimal effect on overall conclusions [32]. We will also exclude duplicate articles.

#### Search strategy.

This scoping review will consider all relevant published, unpublished, and grey literature that meets the inclusion criteria. Following the JBI methodology for scoping reviews, the review will follow a three-step search strategy [30]. It was developed under the guidance of an expert medical librarian with expertise, as recommended by McGowan and Sampson [33].

(1) Preliminary search: An initial exploratory search of PubMed will be conducted to examine key articles related to patient safety culture in the ED and to develop the search strategy. Words from the titles and abstracts of relevant articles and the index terms used to describe them will be analyzed to develop a comprehensive search strategy for PubMed (Table 1).
(2) Comprehensive database search: A structured search will be conducted across multiple databases, including PubMed, the Cumulative Index to Nursing and Allied Health Literature (CINAHL) via EBSCOhost, Web of Science (WOS), Excerpta Medica Database (Embase), Cochrane Library, and the Korean Studies Information Service System (KISS). The search strategy, incorporating all identified keywords and index terms, will be adapted for each included information source.
(3) Reference list and grey literature search: The reference lists of the included articles will be screened for additional sources. Grey literature sources include ProQuest Dissertations & Theses Global, Google Scholar, and websites of international patient safety organizations (e.g., the Agency for Healthcare Research and Quality, Joint Commission, Institute for Healthcare Improvement, and the Korean Patient Safety Reporting and Learning System).

**Table 1:** Search strategy for PubMed.

No.	PCC	Search components	Query
#1	Population	Healthcare workers	“healthcare worker” OR “healthcare provider” OR “healthcare professional”
#2	Concept	Patient safety culture	“patient safety culture” OR “patient safety climate” OR “safety culture” OR “safety climate” OR (patient safety[MeSH Terms])
#3	Context	Emergency department	“emergency department” OR “emergency room” OR “emergency unit” OR (Emergency Service, Hospital[MeSH Terms])
#4	Other	Research conducted since 2004	(2004:2025[pdat])
#5		Research published in English or Korean	(english[Filter] OR korean[Filter])

The search query will be applied as follows: #1 AND #2 AND #3 AND #4 AND #5 in all fields.

The literature search will be limited to articles published after 2004, when the Agency for Healthcare Research and Quality released its original version of the Hospital Survey on Patient Safety Culture to assess hospital patient safety culture [34].

### Stage 3: Study selection

All identified records will be collated and uploaded into EndNote V.21.4 (Clarivate Analytics, PA, USA). Duplicate records will be identified and removed using EndNote’s automated deduplication function, followed by manual review to ensure accuracy. The study selection proceeds in several steps [35]. Based on the inclusion criteria, the initial screening will be conducted independently by two researchers. In this phase, titles and abstracts will be screened and categorized as *included*, *excluded*, or *uncertain*; they will be excluded when they do not fit the Population-Concept-Context. Discrepancies will be resolved through discussion or by consulting a third researcher. Articles marked as included or uncertain in the initial screening will be retrieved in full text, with their citation details imported into the JBI System for Unified Management, Assessment, and Review of Information (JBI SUMARI; JBI, Adelaide, Australia) for study screening [36]. Subsequently, the same two researchers will independently assess whether the full-text articles meet the eligibility criteria according to the review question. If discrepancies arise in the screening results, a third researcher reviews and consults them to reach a consensus. The search results will be fully reported in the final scoping review, including the reasons for excluding full-text articles, and presented in a PRISMA flowchart [37].

### Stage 4: Charting the data

#### Data extraction.

Two researchers will independently extract data from the articles included in this review using a piloted data extraction tool developed by the researchers based on the JBI Manual for Evidence Synthesis [30], which has been piloted. Extracted data will comprise study information, such as author(s), year of publication, aim, inclusion and exclusion criteria, and key results related to the review question, including the participants’ most and least positively perceived dimensions. Details of the draft extraction tool are presented in Table 2. We will also use JBI SUMARI for the data extraction and synthesis. The data extraction tool will be modified and refined as required during the extraction process. Similar to the study selection process, two researchers will independently extract the necessary data. In case of disagreement between researchers regarding data extraction, a third researcher will review and consult them in consensus meetings. This process will continue until consensus is reached among all three researchers.

**Table 2:** Draft data extraction tool.

Categories	Sub-categories	Details
Study information	Author(s)	
	Year of Publication	
	Country of origin	
	Study design	
	Aim(s)	
	Data collection method(s)	
	Data analysis method(s)	
The inclusion and exclusion criteria	Population	Role (position)
		Sample size
	Concept	Definitions
		Instruments
		Dimensions
		Perceptions
		Barriers and facilitators
	Context	Study setting
Key results related to the review question	Outcomes	The most and least positively perceived dimensions
		Research gaps

### Stage 5: Collating, summarizing, and reporting results

#### Data analysis and presentation.

The extracted data will be analyzed and organized thoroughly to align with the objectives of the scoping review. Descriptive analysis will be used to map the data distribution. Content analysis will be conducted to categorize the theoretical frameworks, key definitions of patient safety culture, and methodological approaches used in the included studies. This will help to identify the dominant perspectives and research gaps in the field. Finally, a thematic analysis will focus on the perceptions, barriers, and facilitators of patient safety cultures in the included literature. Among the results, role, sample size, instruments, the most and least positively perceived dimensions, and the study setting will be shown in tabular form or figures. The rest will be presented with a narrative summary to support analysis and synthesis and mapped into three main categories as follows:

(1) Theoretical conceptualization of patient safety culture
(2) Measurement of patient safety culture
(3) Facilitators, barriers of patient safety culture, and policy implications

## Discussion

This scoping review aims to assess the scope, nature, and extent of the literature on the research topic and patient safety culture perceived by HCWs in EDs, map the concept, and identify research gaps in the current literature. The findings of this review will provide valuable guidance to researchers in healthcare services and policymakers for developing interventions to enhance the patient safety culture in EDs.

One of the key strengths of this study is its comprehensive approach, which allows a thorough understanding of the topic. Methodological rigor, which is likely to follow the established guidelines, adds to the credibility of this study. Incorporating perspectives from various stakeholders, including nurses, physicians, and administrative staff, could offer a more holistic view of patient safety culture. Furthermore, the potential impact of this study is significant. The findings will inform policy changes and best practices in EDs, ultimately enhancing the patient safety culture.

Despite these strengths, this review has several potential limitations. One is the exclusion of an assessment of the quality or methodological rigor of the included studies, which may limit their ability to provide definitive conclusions or suggestions for improving clinical practices or policies [38]. We also recognize that excluding papers that were not published in English or Korean may be a limitation of this review. Despite these limitations, we believe that our plans are methodologically rigorous enough to contribute clear conclusions that can answer the review questions, leading to future research, improved clinical practice, and policy-making regarding patient safety.

This study does not require ethical approval because it synthesizes the results of accessible studies without identifiable information. The results of this scoping review will be presented at academic conferences and published in peer-reviewed journals. This scoping review will be conducted in accordance with the JBI’s methodology and the ethical principles outlined in the institutional and journal guidelines on research integrity.

This protocol has been formally registered in the Open Science Framework (https://osf.io/9f7qc), where the search strategy or study progress will be updated based on data availability. Data collection was completed in October 2024, and the results will be summarized and reported by March 2025.

## Supporting information

S1 File PRISMA-ScR checklist. (DOCX)
